# A Biphasic Calcium Sulphate/Hydroxyapatite Carrier Containing Bone Morphogenic Protein-2 and Zoledronic Acid Generates Bone

**DOI:** 10.1038/srep26033

**Published:** 2016-05-18

**Authors:** Deepak Bushan Raina, Hanna Isaksson, Werner Hettwer, Ashok Kumar, Lars Lidgren, Magnus Tägil

**Affiliations:** 1 Department of Orthopedics, Clinical Sciences, Lund, Lund University, Lund, 221 85, Sweden; 2 Department of Biological Sciences and Bioengineering, Indian Institute of Technology Kanpur, Kanpur, 208016, India; 3 Department of Biomedical Engineering, Lund University, Lund, 221 85, Sweden; 4 Department of Orthopedic Surgery, Rigshospitalet, University of Copenhagen, Copenhagen, 2100, Denmark

## Abstract

In orthopedic surgery, large amount of diseased or injured bone routinely needs to be replaced. Autografts are mainly used but their availability is limited. Commercially available bone substitutes allow bone ingrowth but lack the capacity to induce bone formation. Thus, off-the-shelf osteoinductive bone substitutes that can replace bone grafts are required. We tested the carrier properties of a biphasic, calcium sulphate and hydroxyapatite ceramic material, containing a combination of recombinant human bone morphogenic protein-2 (rhBMP-2) to induce bone, and zoledronic acid (ZA) to delay early resorption. *In-vitro*, the biphasic material released 90% of rhBMP-2 and 10% of ZA in the first week. No major changes were found in the surface structure using scanning electron microscopy (SEM) or in the mechanical properties after adding rhBMP-2 or ZA. *In-vivo* bone formation was studied in an abdominal muscle pouch model in rats (n = 6/group). The mineralized volume was significantly higher when the biphasic material was combined with both rhBMP-2 and ZA (21.4 ± 5.5 mm^3^) as compared to rhBMP-2 alone (10.9 ± 2.1 mm^3^) when analyzed using micro computed tomography (μ-CT) (p < 0.01). In the clinical setting, the biphasic material combined with both rhBMP-2 and ZA can potentially regenerate large volumes of bone.

Bone regeneration is one of the most commonly explored areas for tissue engineering^1^. Partly due to a demographic shift towards an older population, there is an increased demand for bone grafts in non-unions, large bone defects in infections, aseptic prosthetic loosening with osteolysis, after tumor surgery and in fragility fractures^2^. In these situations, bone autografts have been used for decades to regenerate bone but the amount of autograft is limited and the harvest of large quantities of autograft is associated with substantial morbidity^3^,^4^,^5^. The other alternative to autografts is the use of allografts. However, their efficacy depends on the donor age as well as tissue banking sources^6^,^7^,^8^, have the risk of disease transmission^9^ and are less efficacious compared to autografts. The increasing demand and the absence of a viable solution for replacing large volumes of bone, poses a scientific challenge that requires new innovative bone graft solutions.

Bone is a combination of both organic and inorganic components. Ceramic, polymer or composite materials have been used to mimic the natural bone, all with the aim to restore bone and improve bone regeneration^10^,^11^. Many osteoconductive scaffolds allow some formation and ingrowth of bone from surrounding tissue, but in large defects it remains a challenge to recruit and differentiate the inducible cells to remodel the bone defect^12^,^13^. Biomaterials designed for bone regeneration therefore are required to induce bone formation at the desired locations. Desired scaffold pre-requisites include optimal physical properties such as sufficient internal space for new bone to grow in with space for the exchange of nutrients and gases, sufficient mechanical stability, the right surface properties and bioresorbability^11^,^14^,^15^. Also biological properties are important, and in particular signaling molecules are required to recruit mesenchymal progenitor cells. These molecules by preference should be included already at the time of the setting of a bone substitute without additional steps.

A number of inorganic bone substitutes have been used clinically^11^. Ceramic materials mimic the inorganic components of bone but their ability to induce bone is limited if they are not used in a supportive local environment or supplied with growth factors that serve as signaling molecules^16^,^17^,^18^. Autografts act as reservoirs of important signaling molecules like the pro-osteogenic proteins from the transforming growth factor - β (TGF- β) family^19^ at the bone defect but locally administered BMP treatment has also been explored^11^,^20^,^21^. In the few randomized clinical non-union and spine fusion series, BMP’s have never been proven to induce bone healing better than autograft^22^,^23^. A possible explanation for this has been a rising insight into their function, and identifying BMP’s as an inducer of not only bone formation but also bone resorption^24^ due to a RANKL-RANK (osteoblast-preosteoclast) interaction leading to increased osteoclastogenesis^25^,^26^,^27^. We have previously shown that it is possible to pharmacologically modulate the excessive bone resorption caused by the use of BMP, without decreasing the increased bone formation, by adding osteoclast-inhibiting^28^,^29^ bisphosphonates^16^,^20^. Bisphosphonates bind to the mineral phase of the bone with strong affinity and when resorbed induce apoptosis of osteoclasts^28^,^30^. Bisphosphonates today are administered systemically^16^,^20^, but there are unwanted side effects of systemic treatment, like reduced bone remodeling^31^, osteonecrosis of the jaw^32^, gastric problems, flu-like symptoms and a low risk of acute renal failure^33^,^34^ and local delivery of these drugs at the site of action is preferable.

The dosage, stability, delivery and release of BMP’s have always been a concern and different carriers and methods have been suggested^35^,^36^,^37^,^38^. One of the most common methods has been soaking the carrier material in a solution containing the protein, which leads to physical absorption of the protein to the material surface^11^,^39^. This method has some limitations. The soaking time is not standardized, which may influence the clinical effect^40^. Moreover, the release kinetics depends on the type of carrier system being used and an optimal carrier system has not been fully developed yet. A few biphasic preset carrier materials have been published recently including porous polymer-inorganic composites^41^. However, these materials have the risk of antigenicity, uncontrolled degradation, and insufficient mechanical strength. Moreover, the non-injectability of these materials requires invasive surgical procedures.

To overcome these drawbacks, we hypothesized that local co-delivery of soluble, carrier free rhBMP-2 and ZA by means of physical entrapment or chemical binding in a ceramic, injectable biphasic carrier (Cerament™ Bone Void Filler)^42^,^43^ can improve the results. The soluble calcium sulphate phase will resorb over time and thus release the osteoinductive protein initiating osteogenic differentiation of mesenchymal progenitors. ZA bound to the material will then protect the newly formed bone from early resorption caused by the addition of rhBMP-2. The material mimics natural bone matrix and have several advantages such as high degree of protein encapsulation, sustained release behavior and improved surgical handling. The biocompatibility and bioresorbability of the biphasic microporous carrier that sets *in situ* makes it suitable as a carrier material with a controlled release of encapsulated or chemically bound additives. The biphasic material is used clinically as bone void filler and consists of hydroxyapatite (HA) and α- hemihydrate and dihydrate calcium sulphate mixed with a radiopaque compound, Iohexol^42^. After injection, the components set *in situ* into a microporous, osteoconductive matrix. The possibility of adding BMP’s or bisphosphonates to the biphasic material has been documented before^44^. ZA is chemically bound to the HA while the rhBMP-2 is physically entrapped within the resorbing calcium sulphate phase of the ceramic carrier. In this first *in-vitro* and *in-vivo* trial, we incorporated both soluble rhBMP-2 and ZA in the ceramic powder and analyzed the release and biological response with an aim to design an osteoinductive tool for large bone reconstructions. We hypothesized that a gradual release of BMP’s from the biphasic carrier could induce osteogenesis while chemically bound ZA will provide protection against premature bone resorption.

## Results

### *In vitro* rhBMP-2 release

**Figure 1:**
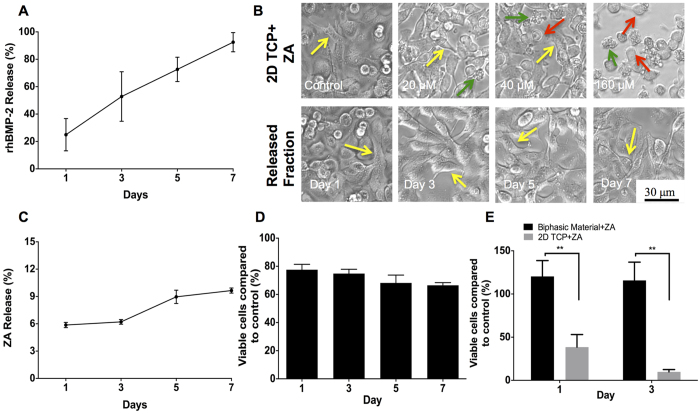
*In-vitro* rhBMP-2 and ZA release kinetics. (**A**) Indicates rhBMP-2 release (%) from the biphasic material over a period of 7-days detected using ELISA. (**B**) Shows the microscopic effect of free ZA (increasing concentrations using tissue culture plates (2D TCP)) and ZA released from the biphasic material (different day fractions) on A549 lung cancer cells (Yellow arrow indicates healthy, epithelial morphology of cells, red arrow points at round, dead cells while green arrow shows floating apoptotic bodies). (**C**,**D**) Fraction of ZA released (%) from the biphasic material over a period of 7-days and the cytotoxicity induced in A549 cells by the released fraction using the MTT assay, respectively. (**E**) Effect of bound and free ZA on A549 cells after seeding the cells directly on the biphasic material alone, in combination with ZA and plastic control treated with free ZA using the MTT assay. **Indicates p < 0.01, ^#^indicates non-significant. Data is expressed as mean ± SD. Scale bar represents 30 μm.

A gradual but constant release of the protein was detected in the supernatants. At day-3, approximately 50% of the protein was detected while nearly 90% of rhBMP-2 was found in the supernatant on 7^th^ day. Figure 1A represents the *in-vitro* release kinetics of rhBMP-2 from the biphasic material.

### *In vitro* ZA release

A dose dependent apoptosis of A549 cells after treatment with ZA was observed in our experiment (Fig. 1B) with higher doses of free ZA causing apoptosis of A549 cells. After plotting a dose vs. cell viability curve, a sustained and minimal release of ZA from the biphasic material was seen. Nearly 6% of ZA originally loaded was released on day 1 that increased to a value of nearly 10% on day 7 (Fig. 1C). Microscopically, no significant differences were seen in the experimental group with cells showing healthy proliferation and morphology when compared to controls (Fig. 1B). Though a very little fraction of ZA was released by the material over 7-days, the cytotoxicity (A549 cells) caused by the released fraction had a decreasing trend with time as seen in Fig. 1D.

### Effect of bound zoledronic acid to the biphasic material

The proliferation of A549 cells on the biphasic material with and without ZA was significantly higher than A549 cells treated with free ZA (p < 0.01) on both day 1 and 3 as shown in Fig. 1E.

### *In vitro* SEM analysis

**Figure 2:**
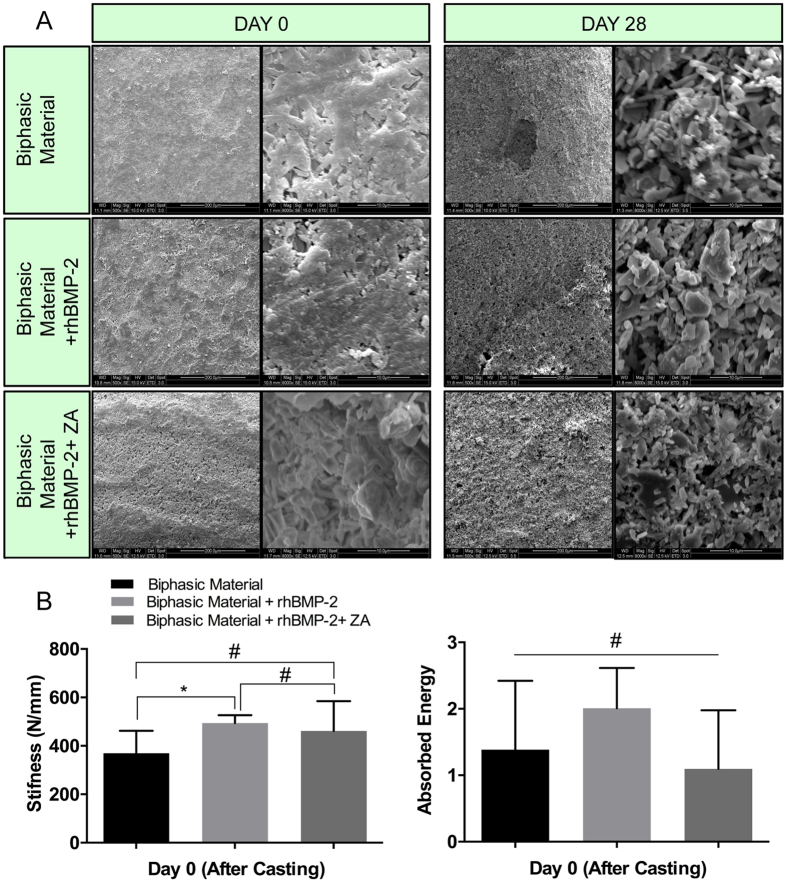
*In-vitro* SEM and mechanical analysis. (**A**) Surface architecture and pore morphology of the biphasic material alone and with rhBMP-2 and rhBMP-2 + ZA was compared after casting (Day 0) and after 28-days of incubation in saline. SEM images on the left panels at Day 0 and day 28 have been captured at 500X while images in the right panel depict high magnification images (8000X). The lower left panel (**B**) shows the stiffness of the biphasic material alone or after addition of rhBMP-2 and ZA. The lower right panel (**B**) indicates the absorbed energy by the samples in different groups. *Indicates p < 0.05, ^#^indicates non-significant. Data is expressed as mean ± SD. n = 5.

The surface as well as the pore distribution across the three groups was similar on day 0 (Fig. 2). High magnification image showed a closed architecture of pores at the time of casting (d = 0). After incubation in saline for 28-days, the samples appeared to be rougher at the surface and an open pore structure was observed. However, the addition of rhBMP-2 or ZA appeared to have no effect on the surface structure of the biphasic material and no differences in the pore structure or surface morphology were observed between the three groups even on day 28 (Fig. 2A).

### *In vitro* mechanical analysis

The stiffness was higher in biphasic material + rhBMP-2 group when compared to the group with the biphasic material alone (p < 0.05). There were no significant differences in stiffness between biphasic material + rhBMP-2 + ZA group compared with biphasic material + rhBMP-2 group or biphasic material alone. All groups showed similar values of absorbed energy (Fig. 2B).

### Assessment of ectopic bone formation

All animals were sacrificed after 4-weeks as described before. One animal in rhBMP-2 and rhBMP-2 + ZA group had to be sacrificed a few days after implantation due to rupture of the skin suture. A total of 10-samples per groups were harvested for rhBMP-2 and rhBMP-2 + ZA groups while 12 samples were obtained in the group containing the biphasic material only.

### Physical assessment of scaffolds at harvest

The samples containing the biphasic material alone were morbid and smaller than the implanted dimension. The samples in the group containing the biphasic material + rhBMP-2 had increased dimensions. On applying a gentle force on the scaffold, blood like fluid oozed out of the scaffolds. The scaffolds belonging to the biphasic material + rhBMP-2 + ZA group were the biggest in dimensions by gross analysis. Moreover, they appeared to be hard and could not be compressed.

### Radiography

**Figure 3:**
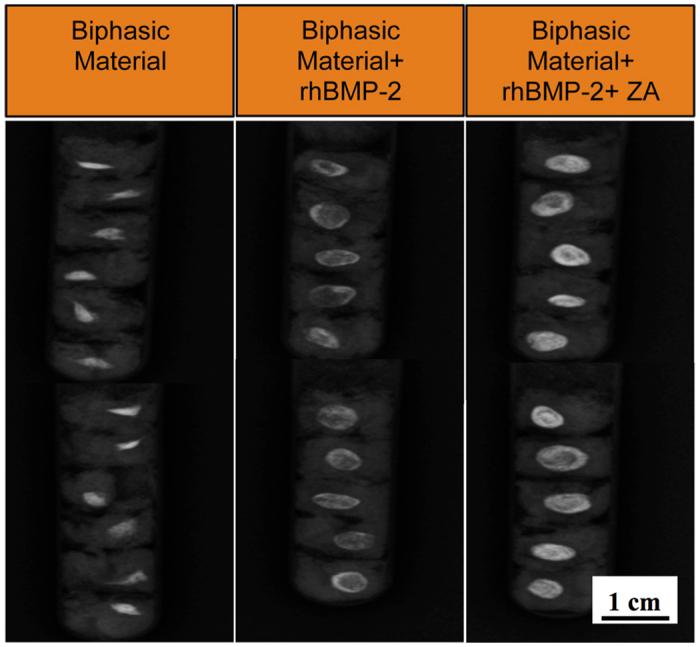
X-ray radiography of implanted samples in the abdominal muscle pouch. X-ray radiographs of biphasic material alone and combined with rhBMP-2 (10 μg) and rhBMP-2 (10 μg) + ZA (10 μg) in the abdominal muscle pouch after 28-days of *in-vivo* implantation. Scale bar represents 1 cm.

Radiographically, the material alone had deformed with respect to its original shape at the time of implantation (Fig. 3). Biphasic material with rhBMP-2 + ZA was most dense radiographically (Fig. 3). Qualitatively, the dimensions of both rhBMP-2 and rhBMP-2 + ZA treated groups based on the radiopaque area appeared larger than the material alone (Fig. 3).

### Micro computed tomography (μ-CT)

**Figure 4:**
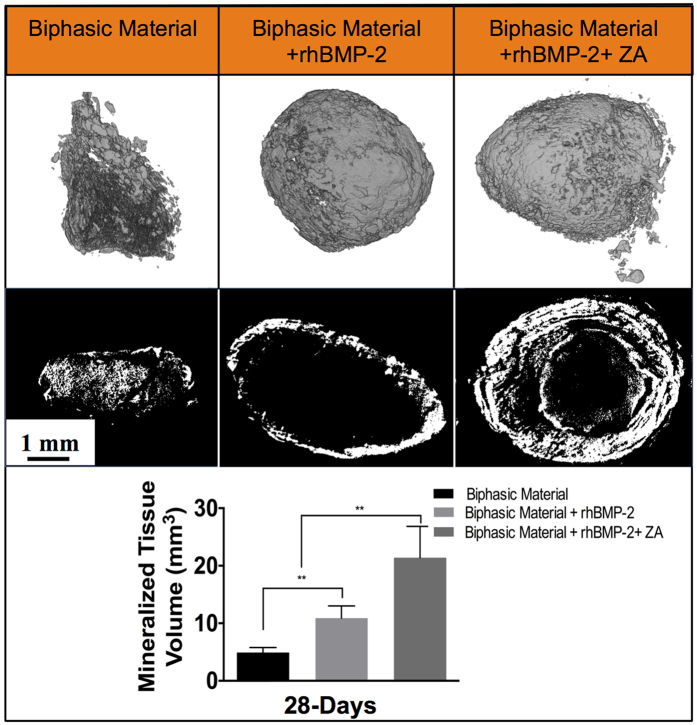
Micro-computed tomography results 28-days post implantation. Images in the top panel represent full 3-D rendering of the samples in the three groups (biphasic material, biphasic material + rhBMP-2 (10 μg) and biphasic material + rhBMP-2 (10 μg) + ZA (10 μg)) while images in the middle panel show sliced 2-D images in the middle of the samples in order to emphasize on the internal content of the samples across different groups. The bottom panel shows the mineralized volume in each group. **Indicates p < 0.01, ^#^indicates non-significant. Data is expressed as mean ± SD. n =  5 for biphasic material + rhBMP-2 and rhBMP-2 + ZA groups and n = 6 for biphasic material alone. Scale bar represents 1 mm.

The biphasic material loaded with a combination of rhBMP-2 + ZA showed both quantitatively and qualitatively a higher amount of mineralized volume (remaining biphasic material + newly formed bone) (21.4 ± 5.5 mm^3^) than the other groups (p < 0.01) (Fig. 4). 3-D and 2-D rendering of the slides shows that the mineralized areas are retained in this group (Fig. 4). The biphasic material + rhBMP-2 showed a higher amount of mineralized volume (10.9 ± 2.1 mm^3^) than the biphasic material alone (Fig. 4). However, the 2-D rendering in the middle panel shows a hollow core in the material treated with only rhBMP-2 (Fig. 4). The mineralized volume was the least in the biphasic material implanted alone (4.9 ± 0.9 mm^3^).

### Histology and histomorphometry

**Figure 5:**
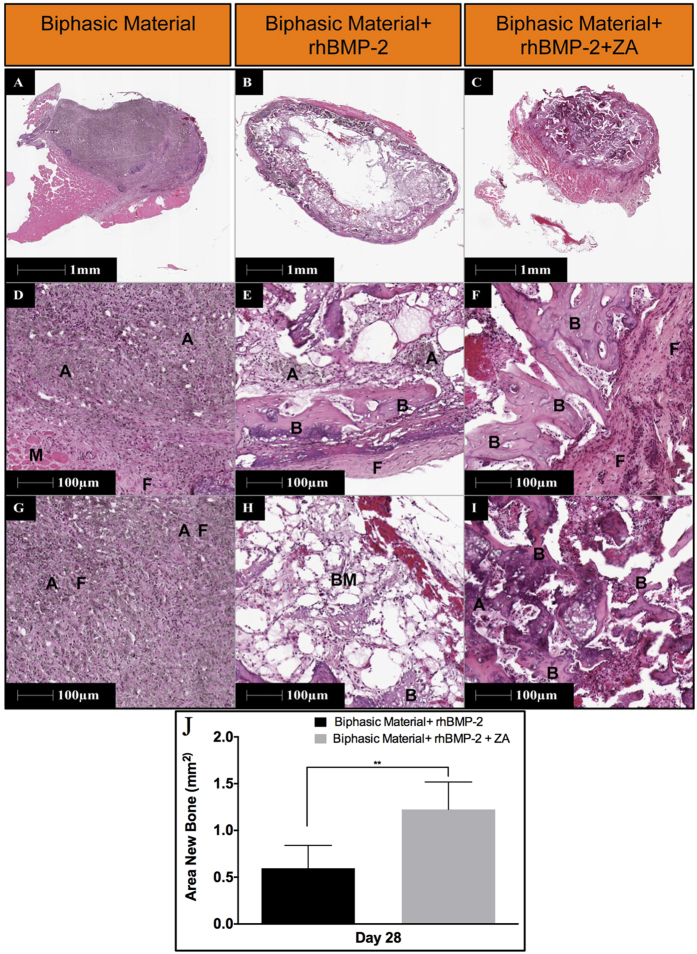
Histological representation and histomorphometric analysis of the samples implanted in the abdominal muscle pouch. Images in top panels provide an overview of the whole sample (12.5X) in the three groups after 28-days of implantation. Images in the middle panel emphasizes on the periphery of the material/bone composite (100X) while images in the bottom panels indicate the innermost tissue/material construct (100X). A indicates apatite, B shows bone, F represents fibrous tissue and “BM” shows bone marrow. Data in (**J**) shows Histomorphometric quantification of bone area across the two groups with bone formation. Data is expressed as mean ± SD. n = 5.

The samples containing only the biphasic material did not show any bone formation. It can be seen as brown to black crystals of HA covered by a layer of muscle and some fibrous tissue, although it was well infiltrated with granular cells (Fig. 5). On the other hand, samples containing rhBMP-2 exhibit bone like morphology in many places (Fig. 5). However, it appears to have undergone osteoclastic resorption characterized by the presence of fatty marrow cells visible in different areas of the scaffold. The group treated with a combination of rhBMP-2 and ZA has developed a cortical shell around the biphasic material with islands of trabecular bone abundantly present all across the scaffold and even in the middle (Fig. 5). Histomorphometrically, significantly higher area of bone formation was seen in the biphasic material combined with rhBMP-2 and ZA when compared to the biphasic material + rhBMP-2 group (p < 0.01).

### Scanning electron microscopy (SEM)

**Figure 6:**
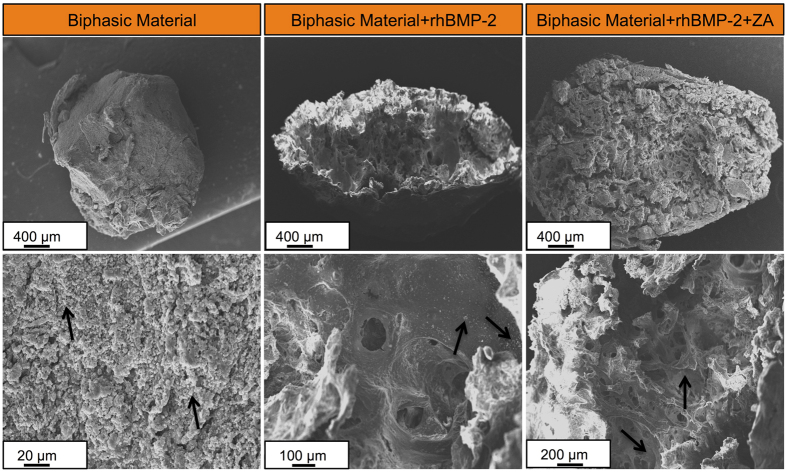
SEM analysis of implanted samples after 28-days of *in-vivo* implantation. Top panel provides a low magnification (50X) overview of samples in the three groups while the images in the lower panels have been taken at comparatively higher magnifications to emphasize on the appearance and surface structure of the bone/material composite. The arrows in lower left and middle panel indicate apatite particles while the arrows in the lower right panel show typical trabecular bone formation on the biphasic material loaded with rhBMP-2 and ZA.

The implanted samples loaded with rhBMP-2 have developed a hollow core in the middle with bone formation on the sides (Fig. 6). Biphasic material treated with rhBMP-2 and ZA appears to be intact and bony ossicles can be seen spread all across the material showing bone progression towards the center of the scaffold (Fig. 6). Typical trabecular morphology was observed even in the middle of the bone/material composite (Fig. 6, right lower panel). Most of the bone/material composite is retained. Only biphasic material appears to have undergone no bone formation and porous material and HA particles can be seen in Fig. 6.

### Mechanical compression

**Figure 7:**
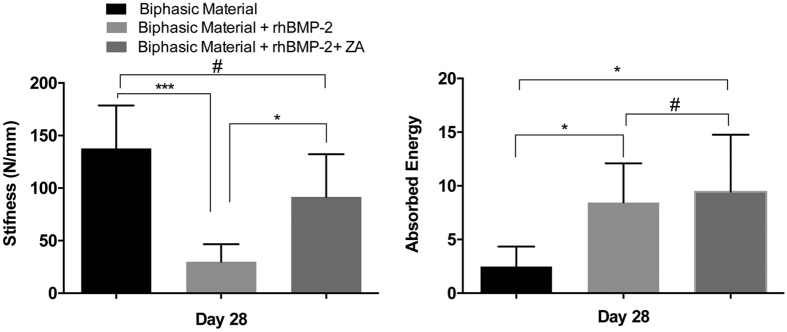
Mechanical testing of implanted samples after 28-days of *in-vivo* implantation. Left panel shows the stiffness of the bone/material composite in the three groups while right panel indicates the absorbed energy across the three groups. *Indicates p < 0.05, ***p < 0.001, ^#^indicates non-significant. Data is expressed as mean ± SD. n = 5.

The stiffness of the biphasic material alone was significantly higher than the group where the biphasic material was treated with rhBMP-2 (p < 0.001) (Fig. 7). No significant differences were found between biphasic material alone and biphasic material + rhBMP-2 + ZA groups. Also the biphasic material + rhBMP-2 + ZA group was significantly stiffer than biphasic material + rhBMP-2 group (p < 0.05). In terms of absorbed energy, the biphasic material combined with rhBMP-2 and rhBMP-2 + ZA absorbed significantly more energy as compared to the material alone (p < 0.05). No significant differences between the rhBMP-2 and rhBMP-2 + ZA groups were seen in terms of absorbed energy.

## Discussion

The osteoblast effect and osteoinductive potential of BMP’s is well established^45^, but the simultaneous osteoclast induction, causing premature resorption of the newly formed callus is less known^20^,^24^,^27^. Although bisphosphonates have primarily been given to increase or maintain bone mineral content in patients with low bone density, in animal models, systemic bisphosphonates can also be used to block the BMP mediated bone resorption and pharmacologically balance the anabolic and anti-catabolic effects of the two drugs^16^,^21^. Further, there is room for improvements regarding the delivery of the protein and BMP’s have been delivered using carboxymethyl cellulose (CMC) or bovine collagen particles^16^ as carriers, or by impregnation of collagen sponges^12^,^39^,^41^,^46^ and other polymers^47^. These carriers have limitations, like the impregnation time that can lead to varying clinical outcomes. Further, the carrier collagen may cause an adverse tissue reaction, mainly due to a local inflammatory reaction. In the present study, we addressed both the premature resorption and the burst release and investigated a one-stage method of delivering the combination of rhBMP-2 and ZA, locally at the site where bone formation is needed. rhBMP-2 and ZA were encapsulated within a ceramic matrix consisting of hydroxyapatite and calcium sulphate. We were able to show a constant but yet high rhBMP-2 release (Fig. 1A), still at the end of 7-days in the current *in-vitro* study. The biphasic nature of the material led to release of the soluble calcium dihydrate sulphate containing the embedded protein. The SEM analysis showed that the samples have more pronounced pores and a rough surface after incubation in saline for 28 days (Fig. 2A). Rough and porous materials have been reported to support osteogenesis^10^,^11^. However, the material may have different release kinetics *in-vivo* enabling a gradual and more extended release of the protein (due to lesser volume of body fluids, hematoma and cellular infiltration around the material). The results from the *in-vitro* release experiment suggested that the protein did not interact with the material by other means than physical entrapment, which can ensure its availability in recruiting and differentiating osteoprogenitor cells. Also, more than 90% of the embedded protein was recovered after 7-days, which also emphasizes high degree of protein encapsulation.

Additionally, the release of ZA from the biphasic material was measured *in-vitro*. Bisphosphonates, in contrast to BMP are most often delivered systemically^16^,^20^, and there are reports that local bisphosphonate treatment may interfere with osteoblastic bone formation^48^, which seems to be possibly tackled by combining it with BMP’s^21^. A few reports state successful co-delivery of BMP’s and ZA by a local scaffold. ZA is known to induce apoptosis in A549 cells in a dose dependent manner^49^ and we used a bioassay for ZA release kinetics *in-vitro*. It is known that bisphosphonates in general, and third generation such as ZA in particular, have high affinity for hydroxyapatite^30^ and we speculate that the concentration of HA in our material led to a strong binding of ZA leaving only a limited amount of unbound ZA behind (Fig. 1B–D). In our model, the strong binding of ZA to the hydroxyapatite is advantageous in retaining high bone turnover. Our results also indicate that bound ZA does not seem to have a cytotoxic effect on A549 cells when seeded directly on the biphasic material mixed with ZA (Fig. 1E). This reaffirms that only a limited amount of ZA is released since no negative effects were observed regarding cell proliferation. A similar release pattern of ZA from a porous collagen-HA scaffold has previously been shown to have a protective effect on pre-osteoblastic cells by the bound ZA^50^. The mechanism is unclear but we speculate that A549 cells do not interact with HA unlike the osteoclasts and thus the negative effects of ZA bound to HA were not seen. In case of free ZA, available for the cells in the culture medium, the drug may enter the cell inducing apoptosis.

No significant delay was observed regarding the setting of the ceramic material which has been reported to be a concern^51^. *In-vitro* mechanical compression (Fig. 2B) showed no reduction in the stiffness and absorbed energy in the groups treated with rhBMP-2 and rhBMP-2 + ZA when compared to biphasic material only, contrary to what has been reported earlier^51^. The *in-vitro* mechanical and SEM analysis of the samples alone or in combination with rhBMP-2 and ZA does not show significant differences between the groups. This strengthens the feasibility of using this biphasic material clinically since the additives do not compromise the surface or mechanical properties of the biphasic material.

The *in-vivo* ectopic bone formation model has been widely used to assess the carrier properties or osteoinductivity of various scaffolds. The doses for rhBMP-2 and ZA in our study were based on previous studies^50^,^52^. In surgery, a one step mixing and delivery of a soluble rhBMP-2 makes this biphasic material an encouraging carrier. No apparent loss in the bioactivity of protein has been observed due to physical entrapment in the set calcium dihydrates and importantly, significant bone formation was seen also with the low doses of rhBMP-2. In a previous study using the ectopic bone formation model, we were able to lower the dose and still observe bone formation at a 2-μg rhBMP-2 added to the biphasic material (Raina DB, 2014, unpublished data). Delivery of low doses of BMP remains an important goal by itself, given the recent report of negative effects with supraphysiological doses presently used^53^.

The *in-vitro* ZA release kinetics is in accordance with the *in-vivo* results. While both rhBMP-2 and rhBMP-2 + ZA treated groups were able to induce bone formation, significant differences were observed. The ectopic bone found using radiography, μ-CT, histological, histomorphometry and SEM analysis are well in accordance and clearly emphasize that the co-delivery of BMP-2 and ZA using the biphasic ceramic material is successful in the current muscle model compared to previous studies^20^,^21^. There are several studies that show systemic bisphosphonate treatment also has similar protection on premature bone resorption^21^,^54^ and it is speculated, based on these studies, that systemic administration of ZA in this study would also have led to similar results as with local delivery. However, the paradigm is shifting towards local ZA treatment keeping in view the side effects of long term systemic bisphosphonate treatment^50^. Significantly higher mineralized volume was found in the biphasic material + rhBMP-2 + ZA group as seen from μ-CT (p < 0.01) (Fig. 4). Addition of rhBMP-2 to the biphasic material doubled the mineralized volume compared to the biphasic material alone while the combination of rhBMP-2 and ZA increased the mineralized volume approximately 4-times compared to the biphasic material alone and 2-times more than the biphasic material combined with rhBMP-2. Histomorphometry results also support the μ-CT data with significantly higher bone formation in biphasic material + rhBMP-2 + ZA group compared to the biphasic material + rhBMP-2 group. (p < 0.01) (Fig. 5). Bone formation was noticed even in the middle of the scaffold (Figs 5 and 6) when rhBMP-2 was combined with ZA. This implies that the scaffold gets porous over time, acting synergistically, enhancing osteoconductivity by dynamic structural remodeling of the material. Without the addition of an anti-resorptive the central part of the scaffold is empty and filled with fibrous tissue or marrow fat. The *in-vivo* results also corroborate with the *in-vitro* rhBMP-2 release, indicating that the carried protein leads to bone formation *in-vivo*. It could be argued that a limitation in the current animal study is the absence of a biphasic material + ZA group. However, from our earlier experiments^20^, the addition of only ZA does not enhance bone formation when compared to the combination of BMP + ZA. Moreover, we are not aware of any reports on ectopic bone formation caused by ZA alone and thus we complied with the 3R’s principle by reducing a group that is known to have little or no impact.

The results from the mechanical analysis after harvest of the *in-vivo* samples provide an insight into the mechanics of the material/bone composite (Fig. 7). Due to the absence of bone in the center of the ceramic samples treated with rhBMP-2 alone (Figs 4, 5, 6), the stiffness of the material was found to be less than the rhBMP-2 + ZA group (Fig. 7). However, the material alone was stiffer even though it appeared to have no bone formation, which maybe due to inherent material properties and the small but dense remaining volume of the biphasic material. This phenomenon was supported by the calculations of the absorbed energy, which demonstrated higher energy absorption for the groups where mineralized tissue had been observed.

The material by itself, without the addition of any signaling molecule did not induce bone formation histologically in this muscle model (Fig. 5). We observed an increase in the mineralized volume (Fig. 4), which may be due to precipitation of hydroxyapatite on the surface of the material due to its composition and irregular surface^10^. This is an expected result in a rodent model^10^,^11^,^55^. As our main aim was to study the carrier properties of the material for rhBMP-2 and ZA by inducing bone at an ectopic location, using the rodent muscle model is well apted for such studies.

**Figure 8:**
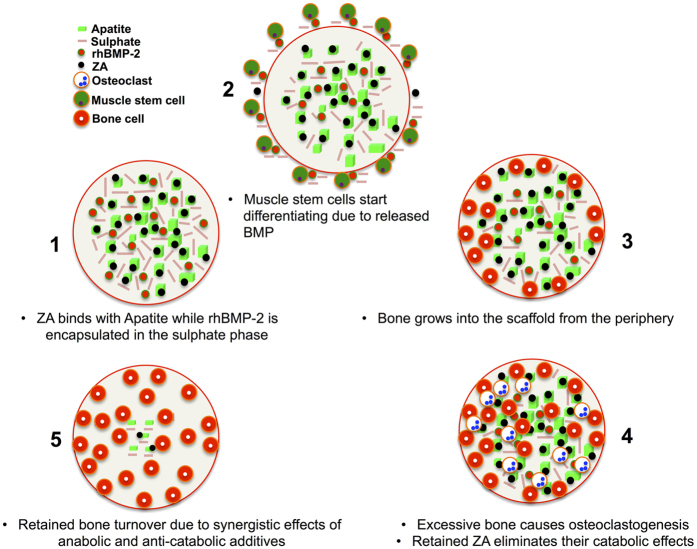
Schematic of rhBMP-2 and ZA delivery from the biphasic material and possible bone formation process. (**1**) indicates a set disc of the biphasic material with ZA bound to HA while rhBMP-2 is encapsulated between the two phases. After *in-vivo* implantation, the material releases sulphate, rhBMP-2 and little ZA as shown in (**2**). Muscle stem cells interact with rhBMP-2 via BMP receptors^56^ and a change in their phenotype occurs leading to their osteogenic differentiation. Subsequently the bone formation approaches inwards into the scaffold. Due to sulphate resorbing over time, the scaffold gets more porous and the bone formation is substantiated by rhBMP-2 as shown in (**3**). After a significant amount of bone is formed, RANKL-RANK (Osteoblast- Osteoclast progenitor) interaction causes osteoclastogenesis as shown in (**4**)^25^. However, due to the presence of ZA, osteoclastic apoptosis occurs^28^ leading to a preserved bone turnover (**5**).

Our results show an easy method with pronounced effect of the co-delivery of ZA and rhBMP-2 and a possible mechanism for the delivery of these bioactive components to induce bone formation in a non-union or in bone regeneration (Fig. 8).

The carrier encapsulates and releases enough rhBMP-2 to induce bone formation in a non-osseous environment by interaction with the BMP-receptor containing surrounding muscle cells^56^. The molecular mechanisms behind the interaction of BMPs with mesenchymal cells have been explained in literature. BMP binds to the receptors of inducible cells causing Smad activation. This leads to transcription of RunX-2 and osteogenic differentiation of the progenitor cells and thus osteoinduction^57^. The combination of the biphasic material with rhBMP-2 provides an osteoinductive tool and we speculate that the molecular mechanisms of BMP mediated osteoinduction remain the same as described above. However, the material just acts as a carrier for the delivery of the osteoinductive molecule and provides a template for bone regeneration. Thus, at this point we do not claim that the biphasic material itself has any osteoinductive properties. Moreover, the bound ZA also enables higher new bone retention by inducing osteoclastic apoptosis^28^, delaying the remodeling of the new-formed bone.

In future studies, we would like to evaluate the osteoinductive potential of the material in large animal models (non-human primates). Moreover, we are evaluating the effect of released BMP and ZA on various progenitor cells to confirm that the molecular mechanism of osteoinduction using the biphasic material and rhBMP-2 follows the conventional molecular pathways that have been described earlier.

Thus in conclusion, the selected combination of HA particles and calcium sulphate ensures sufficient chemical binding of ZA to the HA, while an *in situ* setting calcium sulphate phase, physically incorporating BMP, leads to release of the bioactive protein to the inducible cells. The addition of rhBMP-2 and ZA does not alter the structural properties of the material. The high degree of non-resorbing HA attracting ZA with high affinity is advantageous for local administration of ZA. The results overall suggest that the material can act as a carrier for the co-delivery of rhBMP-2 and ZA with a synergistic effect and it can potentially be used for regeneration of large bone defects.

## Methods

### Study Plan

#### In-vitro

The release of rhBMP-2 and ZA from the biphasic material *in-vitro* was analyzed. Mechanical analysis and SEM imaging was performed to ensure that addition of rhBMP-2 and ZA did not cause dramatic changes in the surface or mechanical properties of the material.

#### In-vivo

In the *in-vivo* study, discs of the biphasic material alone, or containing rhBMP-2 or the combination of rhBMP-2 and ZA were implanted in an abdominal muscle pouch. After harvest, the samples were analyzed using radiography, μ-CT, histology, SEM and mechanical compression test.

### Materials

rhBMP-2 was purchased from Medtronic (Medtronic, Infuse^®^ Bone Graft) and zoledronic acid (Novartis) was purchased from the local pharmacy. The biphasic material, Cerament™ was supplied by Bone Support AB, Lund, Sweden. Alpha modified eagles medium (α-MEM) was purchased from Thermo scientific, U.S.A. Heat inactivated fetal bovine serum (FBS) and MTT reagent was purchased from Sigma Aldrich, Germany. A549 lung cancer cells were kindly provided by Aftab Nadeem (Biomedical center, Lund University). rhBMP-2 ELISA kit was purchased from Abcam, Cambridge, U.K. All other reagents were of high purity.

#### Material preparation

##### *In vitro* rhBMP-2 release

The biphasic material was mixed with rhBMP-2 solution (dissolved in Iohexol and saline) and casted in the shape of cylinders (5 mm diameter, 1.8 ± 0.2 mm height containing 40 μl of ceramic paste) in a sterile polypropylene mold. Each disc contained 2.5 μg of rhBMP-2.

##### Material preparation for *in vitro* ZA release and bound ZA experiment

The biphasic material was mixed with ZA (56.25 μg ZA in 500 mg of ceramic powder), as per the concentrations we have used in a few clinical cases (Hettwer *et al.*, unpublished data) and allowed to set in 24-well polystyrene plates.

##### *In vivo* muscle pouch model

The animals were divided into three groups for the animal experiments, animals receiving biphasic material alone, biphasic material in combination with rhBMP-2 and biphasic material with rhBMP-2 and ZA. The discs were casted in specially designed polypropylene moulds as described earlier. Each cylindrical disc contained 83,33 mg ceramic powder (Calcium Sulphate-60%, Hydroxyapatite-40% by weight), 13,5 μl saline and 22,3 μl of iodine based contrasting agent (Iohexol). The discs in the rhBMP-2 group additionally contained 10 μg of rhBMP-2/disc dissolved in saline, while the discs in rhBMP-2 + ZA group contained 10 μg of rhBMP-2 and 10 ug of ZA/disc dissolved in saline. After all additives were added, the paste was rigorously mixed to homogenize the contents and casted as cylinders in the mould described above in aseptic conditions. After a period of 15 min, the discs were monitored for hardness to record any delays in setting time and the casted discs were stored for further use. Discs from all three groups were also used as unimplanted controls for all evaluation methods.

#### In-vitro rhBMP-2 release

In order to achieve a release kinetics curve, discs of the biphasic material containing rhBMP-2 (described in 2.1.1) were immersed in saline in a low-protein binding tube at physiological pH and incubated at 37 °C for 7 days. At each time-point (Day 1, 3, 5 & 7) the supernatants were collected and stored at −20 °C until assay. After 7-days, the collected supernatants were analyzed using ELISA to determine the concentration of protein in the supernatant.

#### In-vitro ZA release

The analysis of ZA release from the biphasic material was investigated by an indirect method. ZA is known to induce apoptosis of lung cancer cell line A549 in a dose dependent manner^49^. A549 cells were thus used to quantify the release of ZA from the biphasic material *in-vitro*. Various doses of ZA (ranging from 0 μM to 320 μM) were given to 1 × 10^4^, A549 cells/well seeded on 96-well polystyrene plates containing 300 μl of culture medium α-MEM/well (containing 10% FBS by volume). The cells were incubated with ZA for 48 h and the cell viability was assessed using the MTT assay and microscopic visualization. Pre-set discs (described in 2.1.2) were incubated with 0.5 ml of α-MEM without FBS for different time points (1, 3, 5 & 7 days) and supernatants were collected at each time point and stored at 4 °C until the assay at the 7^th^ day. The collected supernatants were finally mixed with 10% (v/v) FBS and added to A549 cells (10^4^/well). The cells were incubated with the supernatants for 48 h and the cell viability was calculated using the MTT assay and cell morphology was analyzed using microscopy. Recorded absorbance was then used to calculate the released drug fraction from the standard curve plotted above.

#### Effect of bound ZA to the biphasic material

ZA was mixed (same concentration as used in 2.3) with the biphasic material and seeded with 10^4^, A549 cells on the material. Material without ZA served as negative control while direct addition of free ZA to A549 cells served as positive controls. The cells were incubated for 24 and 72 h and cell viability was calculated using the MTT assay. The experiment was performed to assess the effect of bound ZA on the proliferation of A549 cells.

#### Scanning electron microscopy (SEM)

SEM was performed to analyze and compare the structure and surface architecture of the biphasic material with or without the addition rhBMP-2 and ZA. The samples were analyzed either after casting or by incubating in saline for 28-days to allow dissolution of the calcium sulphate phase and release of other additives. Later, the samples were dried at 37 °C in a vacuum desiccator for 1 day followed by gold coating. The samples were analyzed using a FEI Quanta, SEM analyzer at an operating voltage of 12.5-15 kV.

#### Mechanical compression test

Compression tests were performed on the samples prepared by following the method described in detail in section 2.1.3. The samples were prepared 24 h before the test. The samples were analyzed on an Instron mechanical analyzer (Instron 8511 load frame, MTS FlexTest 40 Controller, MTS TestSuite Multipurpose Elite Software). A pre load of 1 N was used, followed by loading with 0.1 mm/s until a maximum load of 100 N was reached. Load and displacement data were used to calculate the stiffness of the materials and absorbed energy in all three groups.

#### Surgical Procedure for ectopic bone formation

All discs for *in-vivo* implantation were casted in molds as described above in 4.1.3. A total of 12 Sprague-Dawley rats of 6-weeks age divided into two groups were used. The animals were anaesthetized using a combination of pentobarbital sodium (15 mg/ml) and diazepam (2.5 mg/ml) administered intraperitoneal (I.P). The animals were given intramuscular antibiotic prophylaxis (Streptocilin). Each rat was placed in the dorsal supine position and an approximately 2 cm long mid-line skin incision was made in the abdomen. A muscle pouch was created in the external oblique muscle and the material was placed between two layers of the muscle. The muscle pouch was closed using a non-resorbable 5-0 Ethilon to identify the implants post sacrifice. The skin was sutured using a 5-0 Vicryl bioresorbable suture. All twelve animals received two discs of the biphasic material alone on the left side of the abdominal midline at a minimum distance of 1.5 cm apart. Six of the rats also received two discs of biphasic material + rhBMP-2 on the right side of the abdominal midline, separated from each other by at least 1.5 cm. The remaining 6 animals received discs containing biphasic material + rhBMP-2 + ZA on their right side of the abdominal midline. Animals had access to food and water *ad libitum* and after a period of 4-weeks the animals were sacrificed using an I.P overdose of pentobarbital sodium.

#### Assessment of ectopic bone formation

In order to evaluate the total bone formation in the implanted scaffolds, various techniques were used. All assessments were done after harvesting the samples.

##### Physical assessment of scaffolds at harvest

At the time of sacrifice, all samples were assessed manually by palpating the implant site.

##### Radiography

After sacrifice, all samples were immediately placed in sterile gauze drenched in saline and placed in plastic tubes. All scaffolds were imaged in a similar orientation using a GE Healthcare discovery X-ray machine (CT, USA).

##### Micro computed tomography (micro-CT)

All samples were scanned using an isotropic voxel size of 21 μm (nanoScan, Mediso Medical Imaging Systems, Budapest, Hungary) at an operating voltage of 65 kV, 123 μA current using 360 projections and a RAMLAK filter. Images were reconstructed post hoc to a voxel size of 10 μm (Nucline software, VivoQuant 1.22, inviCRO, Boston, MA, USA). The bone mineral density was calibrated by using two hydroxyapatite phantoms of known densities (0.25 g/cm^3^ and 0.75 g/cm^3^) separated by water and a density of 0.46 g/cm^3^ and above was considered mineralized tissue. The entire sample volume was chosen as the region of interest, and the total mineralized volume in the samples was quantified (including both the hydroxyapatite from the remaining biphasic material and the newly formed bone).

##### Histology and histomorphometry

Five samples from each group were fixed in 4% (v/v) formalin solution for 2 days. The samples were cut in two halves using a surgical scalpel and one half was used for histology while the other half was used for SEM. Histology specimens were decalcified in 10% (w/v) ethylenediaminetetraacetic acid (EDTA) for 10 days at room temperature by constant shaking (Grant-Bio, multi rotator) and the EDTA solution was changed every second day. Post decalcification, samples were dehydrated in increasing ethanol gradient and xylene treatment for 1 h each and embedded in paraffin. The samples were sectioned using a microtome (HM355S, Thermo Fischer Scientific, MA, USA) to a thickness of 5 μm and stained using hematoxylin and eosin. Histomorphometry was performed using HALO v1.92 (Leica Biosystems, histomorphometry software) by selecting the areas of bone formation (bone matrix and osteocytes). The area of bone formation in both groups was then plotted for comparison.

##### Scanning electron microscopy (SEM)

Five undecalcified samples were cut into halves and processed for SEM analysis (Carl Zeiss GmbH, EVO18 Special Edition, Germany). The samples were dehydrated using an increasing ethanol gradient, and the excess muscle tissue around the sample was carefully scraped off under a stereomicroscope. The samples were placed in a vacuum desiccator until imaging and just before imaging the samples were gold coated using a cressignton sputter coater. The samples were analyzed at an operating voltage of 15 kV.

##### Mechanical compression

The remaining 5 samples/group were subjected to mechanical compression using an Instron mechanical analyzer (Instron 8511 load frame, MTS FlexTest 40 Controller, MTS TestSuite Multipurpose Elite Software) connected to a 250 N load cell. Each sample was compressed between two cylindrical metal rods (1 cm diameter). A pre-load of 1 N was applied and the loading rate was 0.1 mm/s until failure. The load and displacement data were used to calculate the stiffness of the implanted materials and the energy absorbed by the implanted scaffolds.

#### Ethical Permission

All animal procedures were approved and performed in accordance with the directives of *Jordbruksverket* (animal ethics and licensing committee), the Swedish regulatory authority for the use of animals for experimental purposes. (Ethical approval number M124-14).

#### Statistical Analysis

Statistical analysis was performed on Prism 6 for Mac OS X (Version 6.0 d, GraphPad Software, Inc., CA, USA). Data is represented as mean ± SD. All analysis were performed using unpaired t-test and p < 0.05 was considered to be statistically significant. All results are expressed in triplicates unless otherwise specified.

## Additional Information

**How to cite this article**: Raina, D. B. *et al.* A Biphasic Calcium Sulphate/Hydroxyapatite Carrier Containing Bone Morphogenic Protein-2 and Zoledronic Acid Generates Bone. *Sci. Rep.* **6**, 26033; doi: 10.1038/srep26033 (2016).
